# The Glucose-Lowering Effect of *Mesembryanthemum crystallinum* and D-Pinitol: Studies on Insulin Secretion in INS-1 Cells and the Reduction of Blood Glucose in Diabetic Rats

**DOI:** 10.3390/nu17010193

**Published:** 2025-01-06

**Authors:** Dahae Lee, Sung Jin Kim, Yea Jung Choi, Young Ho Rho, Tae Seok Kang, Yoon Geol Kim, Ki Sung Kang

**Affiliations:** 1 College of Korean Medicine, Gachon University, Seongnam 13120, Republic of Korea; pjsldh@gachon.ac.kr (D.L.); sungjinkim001@gmail.com (S.J.K.); domdada22@gachon.ac.kr (Y.J.C.); 2 Ilivbio Co., Ltd., Seoul 06531, Republic of Korea; ilivbio@naver.com (Y.H.R.); ilivbio_rnd@naver.com (T.S.K.); ilivbio_im@naver.com (Y.G.K.)

**Keywords:** *Mesembryanthemum crystallinum*, D-pinitol, insulin, IRS-2, PDX-1

## Abstract

**Background:** Ice plant (*Mesembryanthemum crystallinum*) is a vegetable with various therapeutic uses, one of which is its ability to prevent diabetes. The present study examined the insulin secretion effect related to the mechanism of action of ice plant extract (IPE) and its active compound D-pinitol in a rat insulin-secreting β-cell line, INS-1, as well as in diabetic rats. **Methods**: The glucose-stimulated insulin secretion (GSIS) test and Western blotting were used to measure GSIS. The glucose-stimulated index (GSI) and expression levels of insulin-related pathway factors, including insulin receptor substrate-2 (IRS-2), phosphoinositide 3-kinase (PI3K), Akt, and pancreatic and duodenal homeobox-1 (PDX-1), were measured in INS-1 cells. **Results**: The results showed that the GSI values were found to be 8.17 ± 0.22 and 12.21 ± 0.22 for IPE (25 μg/mL) and D-pinitol (100 μM), respectively. GSI values increased statistically significantly. In addition, IPE and D-pinitol upregulated the expression of insulin-related pathway factors. These findings indicate that insulin secretion was significantly stimulated by IPE and D-pinitol in the INS-1 cells, partly by upregulating the expression of IRS-2, PI3K, Akt, and PDX-1. Additionally, IPE administration significantly reduced excessive weight gain and improved glucose tolerance by decreasing the OGTT-AUC. It demonstrated liver-function-improving and lipid-lowering effects by reducing serum alanine aminotransferase (ALT), serum aspartate aminotransferase (AST), triglyceride levels, and total cholesterol levels. Mechanistically, IPE enhances insulin signaling by increasing insulin receptor substrate 1 (IRS-1) phosphorylation and improving glucose metabolism and insulin sensitivity. **Conclusions**: These results offer important new information on the potential of D-pinitol and IPE as functional foods for improving insulin secretion and managing metabolic dysregulation associated with diabetes.

## 1. Introduction

Ice plant (*Mesembryanthemum crystallinum*) is a plant that grows natively in Southern Africa, and is capable of withstanding dryness and salt. It is a facultative halophyte recognized for its potential efficacy in preventing lifestyle-related diseases. The possible biological activity of ice plant in preventing and treating metabolic disorders such as diabetes has been acknowledged [1]. Ice plant has drawn much interest as it contains a broad and diverse range of natural compounds with various biological and pharmacological effects [2].

Numerous investigations have revealed that polyols such as D-pinitol are abundant in ice plant. Several studies have demonstrated that D-pinitol is present at the highest levels among cyclitol molecules, whereas chiro- and myo-inositol contents are comparatively low [2,3]. D-pinitol inhibits rat breast carcinogenesis [4] and exhibits antidepressant effects [5]. In addition, the anti-inflammatory and antioxidant qualities of D-pinitol have been extensively investigated [6]. In numerous studies on diabetes, in rats with diabetes caused by streptozotocin, D-pinitol has been demonstrated to increase the insulin sensitivity of insulin target tissues. [7,8,9]. Studies have shown that insulin secretion increases in diabetic animal models following administration of ice plant extract (IPE) and its active compound, D-pinitol [6,8,10,11,12]. However, the mechanisms by which IPE and D-pinitol can affect glucose-stimulated insulin secretion (GSIS) in vitro have not yet been investigated. Therefore, the present study was designed to evaluate the effectiveness of IPE and D-pinitol on glucose-stimulated insulin secretion (GSIS) in vitro.

Diabetes is a group of metabolic diseases characterized by high blood glucose levels. It occurs when the body cannot properly produce or use insulin, a hormone released by pancreatic cells in response to changes in plasma glucose levels. There are two main types of diabetes. Type 1 diabetes is an autoimmune disorder whereby the body’s immune system attacks and destroys insulin-producing beta cells in the pancreas. Type 2 diabetes (T2D) occurs when the body becomes resistant to insulin or when the pancreas cannot produce enough insulin to maintain normal blood glucose levels [13]. T2D is mostly treated with insulin, oral medications, and lifestyle modifications. The goal of the drugs currently used to treat diabetes is to enhance the amount of insulin released by pancreatic cells, improve the insulin sensitivity of insulin target tissues, or both [14]. Available medications for T2D include metformin, sulfonylureas, DPP-4 inhibitors, SGLT-2 inhibitors, and GLP-1 receptor agonists. Despite the development and application of insulin and the availability of contemporary anti-diabetic medications, unexpected side effects of already-existing drugs can significantly interfere with treatment, prompting researchers to explore more potent plant-based medications as an alternate course of treatment for diabetes [15,16].

Insulin secretion was found to be impaired following glucose injection by the homozygous disruption of pancreatic and duodenal homeobox-1 (PDX-1) in a mouse model and in human patients. PDX-1 is an essential insulin transcription factor that contributes to insulin secretion [17]. In addition, for the regulation of pancreatic cell neogenesis and differentiation, PDX-1 is essential [18]. Accumulated evidence has identified PDX-1 as a potential regulator of insulin secretion. To the best of our knowledge, no prior studies have reported on factors impacting the PDX-1 pathway.

This work is the first to clarify the molecular mechanism via which ice plant’s primary active ingredient, D-pinitol, increases insulin secretion in pancreatic cells, mainly through its involvement in the PDX-1 pathway. Additionally, based on the physiological effects of D-pinitol, this study assessed the glucose-reducing effect of ice plant in diabetic rats.

## 2. Materials and Methods

### 2.1. Plant Material

Ice plant (*M. crystallinum*) was acquired from JS Farmer Co., Ltd. (Jinju, Republic of Korea), and identified by DOWGENE Co., Ltd. (Seoul, Republic of Korea). The leaves and stems of *M. crystallinum* were collected on 24 February, then washed and dried.

### 2.2. Preparation of M. crystallinum

The ice plant extract was manufactured by S&D Co., Ltd. (Cheongju, Republic of Korea). *M. crystallinum* was extracted with ethanol, and the extract was concentrated. After mixing in dextrin relative to the solid content of the concentrate, the concentrate was dried to manufacture ice plant extract (IPE).

### 2.3. Analysis of D-Pinitol in IPE

**Figure 1:**
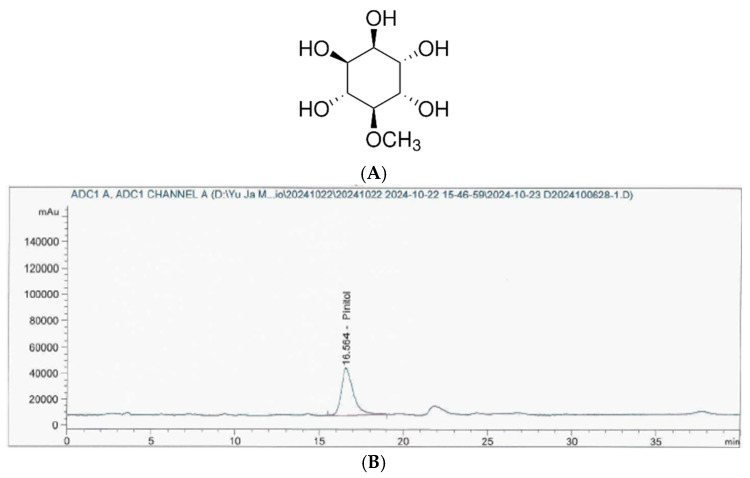
Chemical structure of D-pinitol and HPLC chromatogram of IPE. (**A**) Chemical structure of D-pinitol. (**B**) HPLC chromatogram of IPE.

The Korea Health Functional Food Association (Seongnam, Republic of Korea) analyzed the D-pinitol content in the IPE (Figure 1). Briefly, HPLC analyses were conducted using an Agilent 1260 Infinity pumping system (Santa Clara, CA, USA) equipped with an Alltech 3300 evaporative light-scattering detector (ELSD, Milwaukee, WI, USA) and a YMC-Pack Polyamine II column (25 cm × 4.6 mm, 5 µm (YMC, Kyoto, Japan), flow rate 1.0 mL/min).

### 2.4. Cell Culture

RPMI-1640 medium (Cellgro, Manassas, VA, USA) was used to grow INS-1 cells (Biohermes, Shanghai, China), a rat insulin-secreting β-cell line. The composition of the cell culture medium and cell culture conditions were based on a previous study [19].

### 2.5. Cell Viability Assay

To ascertain the non-toxic concentration ranges of IPE and D-pinitol for a 24 h period, INS-1 cells seeded in 96-well plates were assessed using an Ez-Cytox cell viability detection kit (Daeil Lab Service Co., Seoul, Republic of Korea). Using a microplate reader (PowerWaveXS; Bio-Tek Instruments, Winooski, VT, USA), the absorbance of the culture media of INS-1 cells treated for 24 h in the presence or absence of IPE and D-pinitol was determined at 450 nm.

### 2.6. GSIS Assay

A GSIS assay examined insulin secretion in INS-1 cells treated with IPE and D-pinitol for one hour or left untreated in Krebs–Ringer bicarbonate HEPES buffer (KRBB). The composition of KRBB was based on previous research [20]. The basal and stimulant glucose concentrations were 2.8 and 16.7 mM, respectively. The KRBB of the untreated and treated cells was obtained by centrifugation, and GSIS was determined using a rat insulin ELISA kit (Gentaur, Shibayagi Co., Ltd., Shibukawa, Gunma, Japan), following the manufacturer’s instructions. The glucose stimulation index (GSI) was determined as the ratio of the insulin level at the basal glucose concentration to the insulin level at the stimulating glucose concentration.

### 2.7. Western Blot Analysis

Total protein was extracted using a radioimmunoprecipitation assay (RIPA) buffer (Cell Signaling Technology, Danvers, MA, USA). A sodium dodecyl-sulfate polyacrylamide gel electrophoresis (SDS-PAGE, 10%) gel was used to separate equal amounts (30 μg/lane) of total cellular protein, which were then transferred onto a nitrocellulose membrane (BioRad, Hercules, CA, USA). Using primary antibodies against P-IRS-2 (Ser731), IRS-2, P-IRS-1, IRS-1, P-PI3K, PI3K, P-Akt (Ser473), Akt, PDX-1, and glyceraldehyde 3-phosphate dehydrogenase (GAPDH) (Cell Signaling), polyvinylidene fluoride (PVDF) membranes were incubated at 4 °C overnight, and then incubated with horseradish peroxidase-conjugated anti-rabbit secondary antibodies (Cell Signaling Technology) and with an enhanced chemiluminescence reagent (GE Healthcare UK Limited, Buckinghamshire, UK) for protein visualization using a chemiluminescence system (FUSION Solo, PEQLAB Biotechnologie GmbH, Erlangen, Germany).

### 2.8. Animal Care and Diabetes Induction

Male Sprague Dawley rats (five weeks old) were purchased from BioLink and acclimated to the housing facility for one week, with free access to food and water. The rats were fed a high-fat diet (HFD, D12492, 60% kcal from fat; Research Diet Inc., New Brunswick, NJ, USA). After one week, diabetes was induced by intraperitoneal injection of streptozotocin (STZ; Sigma, Livonia, MI, USA, 30 mg/kg in 0.1 M citric acid buffer, pH 4.5) at a dose of 1 mL/kg, administered twice with a one-week interval. Following induction, the rats were grouped according to their blood glucose levels measured from tail vein samples, with 8 rats per group. Six treatment groups (comprising 8 individuals each) were used: (1) normal diet control group (ND): ND and administration of sodium carboxyl methyl cellulose (CMC) solution; (2) HFD control group: HFD after administration of the CMC solution; (3) positive control group: HFD and administration of metformin (MT) at 250 mg/kg; (4) experimental group: HFD and administration of IPE at 100 mg/kg; (5) experimental group: HFD and administration of IPE at 200 mg/kg; and (6) experimental group: HFD and administration of IPE at 400 mg/kg. All treatment compounds were dissolved in CMC solution and administered orally, once daily, for 9 weeks. Body weight was monitored weekly throughout the study. At the end of the experiment, the animals were euthanized, and epididymal fat was collected for weight measurements. This study was approved by the Institutional Animal Care and Use Committee of Gachon University (GU1-2024-IA0027-00, approved on 15 July 2024).

### 2.9. Oral Glucose Tolerance Test (OGTT)

Oral glucose tolerance tests (OGTTs) was performed in the 9th week after diabetes induction. Before testing, the rats were fasted for 16 h, and glucose (D-(+)-Glucose, Sigma, USA) was administered orally at 2 g/kg. Blood samples were collected from the tail vein at 0, 30, 60, and 120 min, and glucose levels were measured using a blood glucose monitoring system (Accu Check, Mannheim, Germany). The area under the curve (AUC) for the OGTT was calculated using the GraphPad Prism 5.00 software.

### 2.10. Biochemical Assay

The animals were euthanized after fasting for 12 h, and blood samples were collected from the abdominal aorta. The samples were centrifuged at 2000× *g* for 15 min at 4 °C to obtain serum stored at −70 °C for further analysis. Serum aspartate aminotransferase (AST), alanine aminotransferase (ALT), triglyceride (TG), total cholesterol (TC), and glucose levels were measured at a specialized testing facility (GENIA, Seongnam, Republic of Korea). Insulin levels were determined using a rat insulin ELISA kit, following the manufacturer’s instructions.

### 2.11. Statistical Analysis

Statistical significance was determined using one-way analysis of variance (ANOVA) and multiple comparisons with Bonferroni correction. *p* was set at *p* < 0.05, indicating statistical significance. All the analyses were performed using SPSS Statistics ver. 19.0 (SPSS Inc., Chicago, IL, USA).

## 3. Results

### 3.1. Effect of IPE and D-Pinitol on GSIS

**Figure 2:**
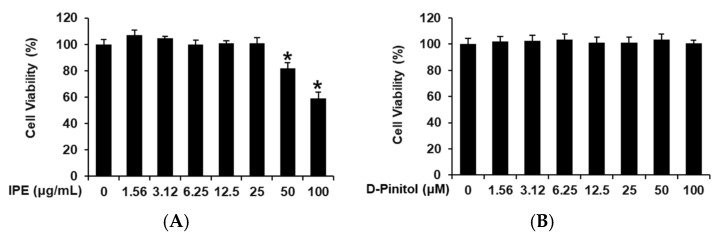
The effects of ice plant extract (IPE) and D-pinitol on the viability of INS-1 cells. The effects of (**A**) IPE and (**B**) D-pinitol on the viability of INS-1 cells after 24 h of incubation, compared to the control (0 μg/mL or 0 μM), determined by cell viability assays; *n* = 3 independent experiments, * *p* < 0.05, Kruskal–Wallis non-parametric test. The data are presented as the mean ± SEM.

**Figure 3:**
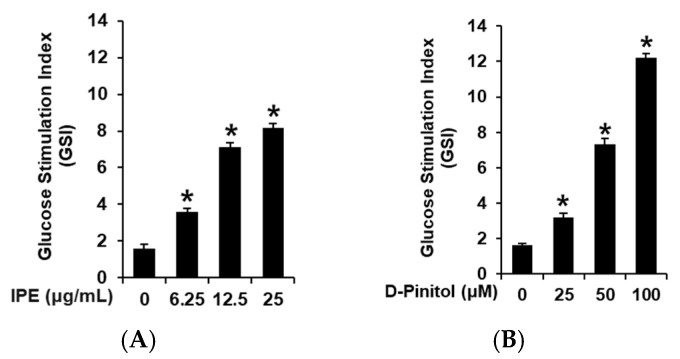
The effects of ice plant extract (IPE) and D-pinitol on glucose-stimulated insulin secretion (GSIS) in INS-1 cells. The effects of (**A**) IPE and (**B**) D-pinitol on GSIS in INS-1 cells after one hour of treatment, compared to the control (0 μg/mL or 0 μM), determined using GSIS assays. The GSISs are compared as the fold-stimulation of the glucose-stimulated index (GSI, 16.7 mM glucose over 2.8 mM glucose for one hour); *n* = 3 independent experiments, * *p* < 0.05, Kruskal–Wallis non-parametric test. The data are presented as the mean ± SEM.

Non-toxic doses of IPE and D-pinitol were determined using an Ez-Cytox cell viability assay in INS-1 cells. IPE showed toxicity at 50 and 100 μg/mL (Figure 2A). D-Pinitol showed no toxic effect at concentrations from 1.56 to 100 μM (Figure 2B). IPE (6.25, 12.5, and 50 μg/mL) and D-pinitol (25, 50, and 100 μM) were used in the GSIS assay. As shown in Figure 3A, the GSI values increased significantly with an increasing concentration of IPE. The GSI values were found to be 3.61 ± 0.18, 7.13 ± 0.22, and 8.17 ± 0.22 for IPE at 6.25, 12.5, and 25 μg/mL, respectively. As shown in Figure 3B, the GSI values increased significantly with an increasing concentration of D-pinitol. The GSI values were found to be 3.19 ± 0.24, 7.31 ± 0.34, and 12.21 ± 0.22 for D-pinitol at 25, 50, and 100 μM, respectively. However, further studies are needed to evaluate the mechanism underlying the improvement in GSIS after treatment with IPE and D-pinitol.

### 3.2. Effects of IPE and D-Pinitol on the Protein Expression of IRS-2, PI3K, Akt, and PDX-1

**Figure 4:**
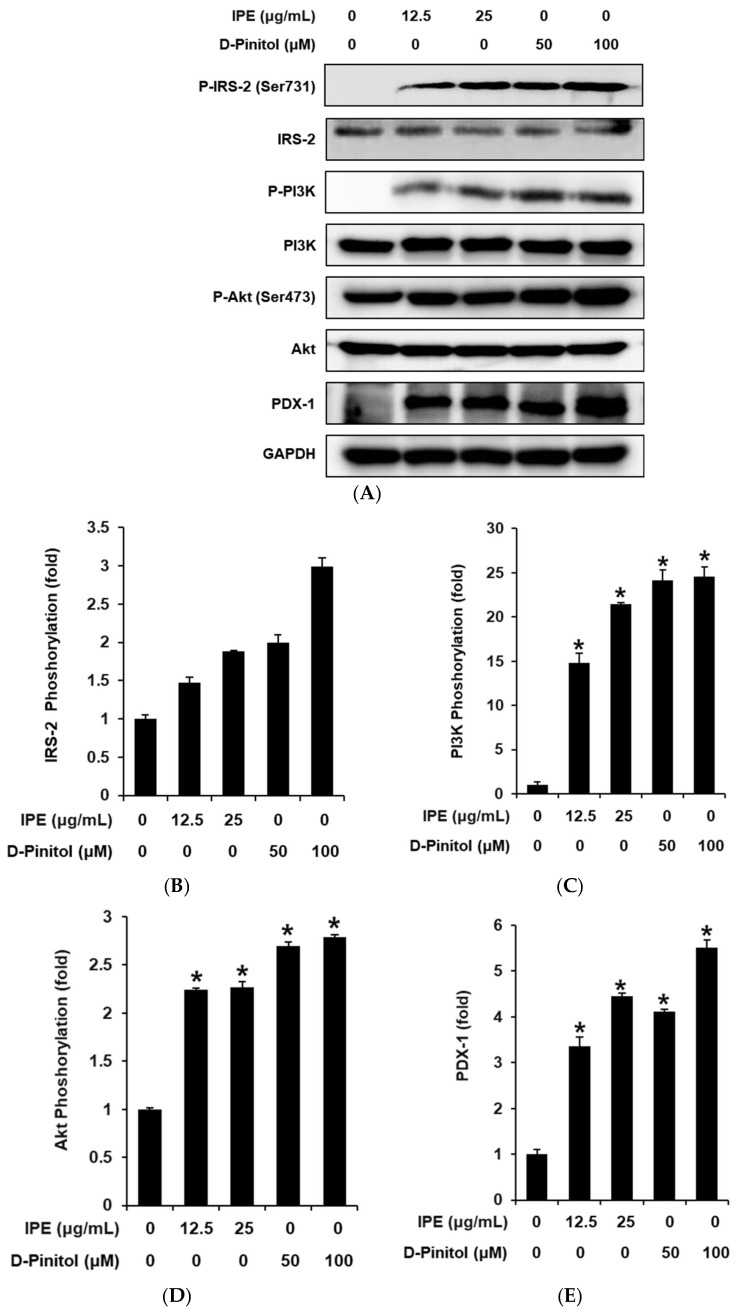
Effects of IPE and D-pinitol on the expression levels of the phosphorylation of insulin receptor substrate-2 (P-IRS-2), IRS-2, phosphorylation of phosphatidylinositol 3-kinase (P-PI3K), PI3IK, phosphorylation of Akt (P-Akt), Akt, and pancreatic and duodenal homeobox-1 (PDX-1) proteins in INS-1 cells. (**A**) Protein expression levels of P-IRS-2 (Ser731), IRS-2, P-PI3K, PI3K, P-Akt (Ser473), Akt, PDX-1, and glyceraldehyde 3-phosphate dehydrogenase (GAPDH) in INS-1 cells treated with IPE (12.5 and 25 μg/mL) or untreated, and those treated with D-pinitol (50 and 100 μM) for 24 h. (**B**–**E**) Each bar graph presents the densitometric quantification of Western blot bands. * *p* < 0.05 compared to the control (0 μg/mL or 0 μM).

We measured the protein levels of IRS-2, PI3K, Akt, and PDX-1 to evaluate their roles in the effects of IPE and D-pinitol on GSIS. Treatment with IPE (12.5 and 25 μg/mL) and D-pinitol (50 and 100 μM) increased the protein expression levels of PDX-1 compared to untreated controls. PDX-1 is mediated by an upstream PI3K/Akt signaling pathway. Treatment with IPE (12.5 and 25 μg/mL) and D-pinitol (50 and 100 μM) increased the protein expression levels of P-PI3K and P-Akt (Ser473) compared to untreated controls. PI3K/Akt signaling is facilitated by IRS-2, an upstream activator. Treatment with IPE (12.5 and 25 μg/mL) and D-pinitol (50 and 100 μM) increased the protein expression levels of IRS-2 compared to untreated controls (Figure 4).

### 3.3. Effect of IPE Administration on Changes in Body Weight in HFD/STZ-Induced Diabetic Rat Model

**Figure 5:**
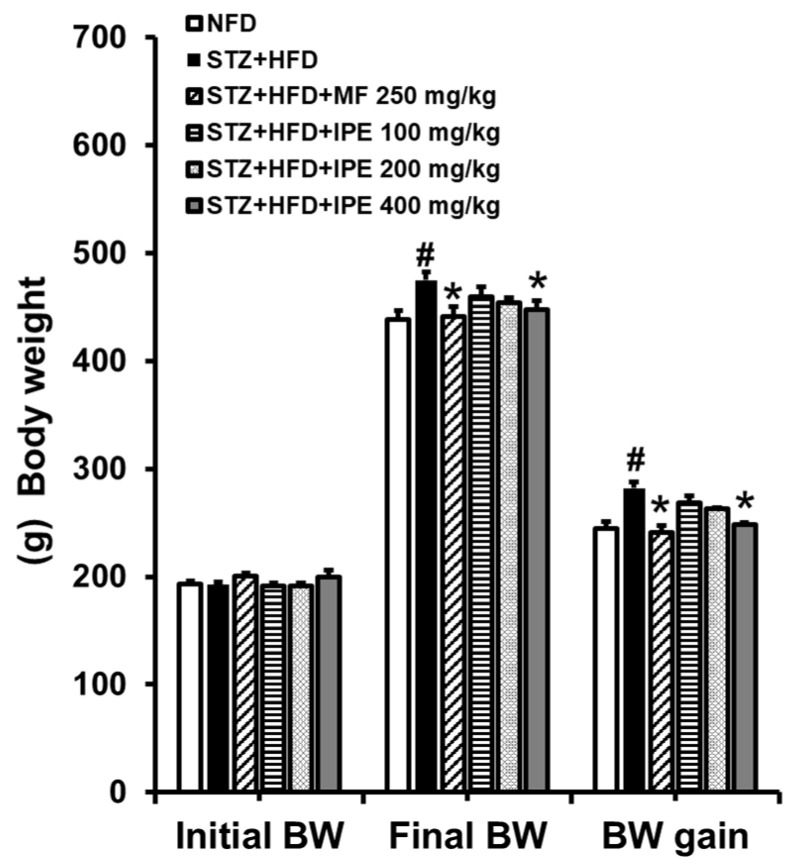
Effect of IPE on body weight (BW) of high-fat diet (HFD)/streptozotocin (STZ)-induced diabetic rats. NFD, normal-fat diet; STZ + HFD, streptozotocin + high-fat diet; STZ + HFD + MF, streptozotocin + high-fat diet + diabetic control + metformin. Values are expressed as means ± SEM (*n* = 8). ^#^ *p* < 0.001 compared with the NFD group. * *p* < 0.001 compared with the STZ/HFD group.

On the first day of IPE administration, no significant differences in body weight were observed between the experimental groups. The initial body weights were as follows: normal-fat diet (NFD): 193.01 g; STZ+ high-fat diet (HFD): 192.64 g; streptozotocin (STZ) + HFD + metformin (MF) (250 mg/kg): 200.44 g; STZ + HFD + IPE (100 mg/kg): 191.30 g; STZ + HFD + IPE (200 mg/kg): 191.69 g; and STZ + HFD + IPE (400 mg/kg): 199.59 g (Figure 5). However, by the final administration day, the STZ + HFD group showed the most significant increase in body weight, with a rise of +282.46 g (final weight: 475.10 g). In contrast, the STZ + HFD + metformin (250 mg/kg) group exhibited the smallest increase, gaining +241.20 g (final weight: 441.64 g). Notably, among the IPE-treated groups, the STZ + HFD + IPE (400 mg/kg) group demonstrated a more modest weight gain of +248.39 g (final weight: 447.98 g), lower than the gains observed in the STZ + HFD + IPE (200 mg/kg) group (+262.83 g, final weight: 454.51 g) and the STZ + HFD + IPE (100 mg/kg) group (+268.45 g, final weight: 459.75 g). Additionally, all of the IPE-treated groups showed statistically significant differences in body weight compared to baseline levels on the first day of administration, reflecting the dose-dependent effect of IPE on mitigating weight gain.

### 3.4. Effect of IPE on Oral Glucose Tolerance Test

To evaluate the effect of IPE on oral glucose tolerance, diabetic rats were orally administered a high dose of glucose. Blood glucose levels were measured at 0, 30, 60, and 120 min, and the area under the curve (AUC) for the oral glucose tolerance test (OGTT-AUC) was calculated (Table 1). Compared to the NFD group (320.0 ± 18.4), the HFD/STZ group showed a significant increase in OGTT-AUC (383.5 ± 27.9). However, the STZ + HFD + MF 250 mg/kg group (355.0 ± 22.4) and the STZ + HFD + IPE 400 mg/kg group (366.4 ± 32.3) exhibited significantly reduced OGTT-AUC values compared to the HFD/STZ group. These results indicate that high doses of IPE improved glucose tolerance in diabetic rats.

### 3.5. Effect of IPE on Serum AST, ALT, TG, and TC Levels

**Figure 6:**
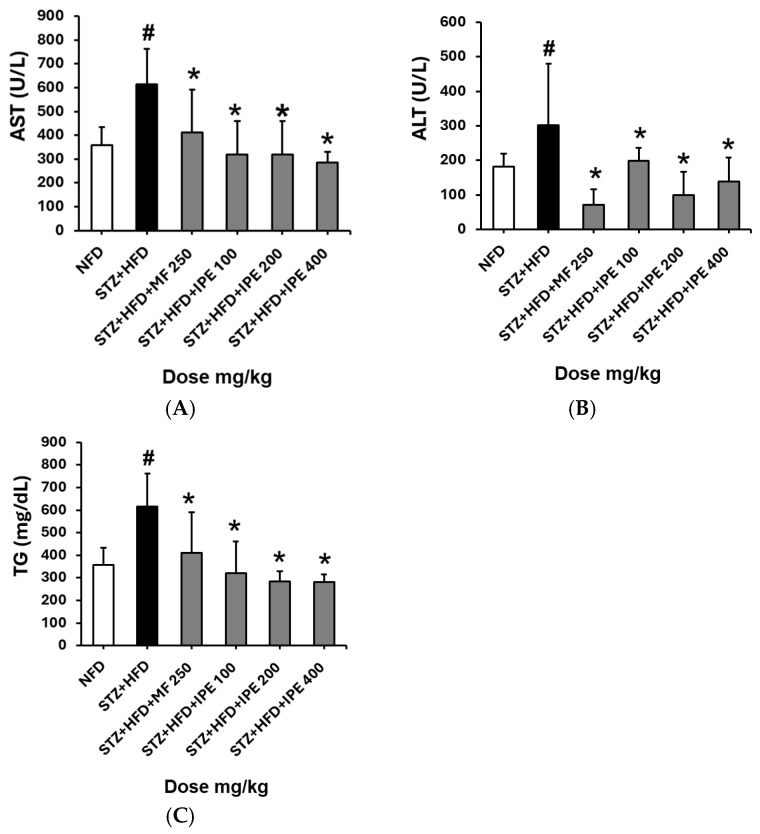
Effect of IPE on aminotransferase (AST), alanine aminotransferase (ALT), and triglyceride (TG) serum levels in high-fat diet (HFD)/streptozotocin (STZ)-induced diabetic rats. (**A**) Effect of IPE on AST. (**B**) Effect of IPE on ALT. (**C**) Effect of IPE on TG. NFD, normal-fat diet; STZ + HFD, streptozotocin + high-fat diet; STZ + HFD + MF, streptozotocin + high-fat diet + diabetic control + metformin. Values are expressed as means ± SEM (*n* = 8). ^#^ *p* < 0.001 compared with the NFD group. * *p* < 0.001 compared with the STZ + HFD group.

In the STZ + HFD group, ALT and AST activities were significantly elevated, recorded as 615 ± 147.8 U/L and 302.3 ± 177.8 U/L, respectively (Figure 6). The positive control group treated with metformin (250 mg/kg) showed reduced ALT and AST levels of 411.1 ± 180.0 U/L and 72.5 ± 43.3 U/L, respectively. Among the IPE-treated groups, the high-dose IPE group (400 mg/kg) exhibited the most significant reduction in ALT and AST activities, measured as 248.8 ± 44.9 U/L and 139.5 ± 68.8 U/L, respectively.

For serum triglyceride levels, the STZ + HFD group exhibited a substantial increase to 256.6 ± 7.17 mg/mL compared to the NFD group (57.47 ± 9.82 mg/mL). The positive control group showed reduced triglyceride levels relative to the STZ + HFD group. Among the IPE-treated groups, the high-dose IPE group (400 mg/kg) showed a significant decrease in serum triglyceride levels to 280.8 ± 34.2 mg/mL, demonstrating the efficacy of IPE in improving lipid levels.

### 3.6. Effect of IPE on Blood Glucose and Insulin Levels

**Figure 7:**
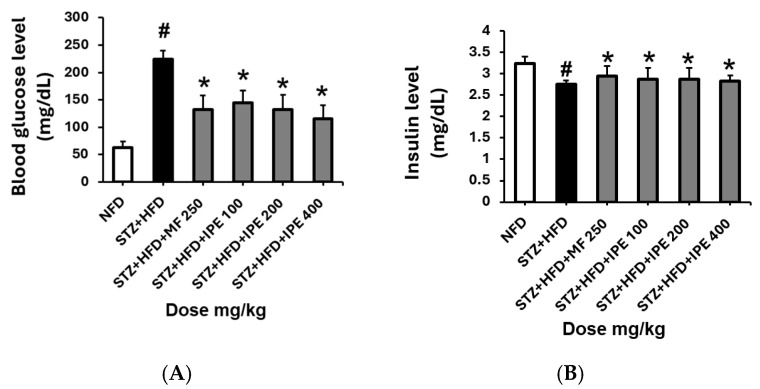
Effect of IPE on blood glucose and insulin levels in high-fat diet (HFD)/streptozotocin (STZ)-induced diabetic rats. (**A**) Effect of IPE on blood glucose level. (**B**) Effect of IPE on insulin level. NFD, normal-fat diet; STZ + HFD, streptozotocin + high-fat diet; STZ + HFD + MF, streptozotocin + high-fat diet + diabetic control + metformin. Values are expressed as means ± SEM (*n* = 8). ^#^ *p* < 0.001 compared with NFD group and * *p* < 0.05 compared with STZ + HFD group.

At week 9, the fasting blood glucose levels in the HFD/STZ group (224 ± 15.7 mg/dL) were significantly higher than those in the NFD group (62.5 ± 11.6 mg/dL). In contrast, the positive control group exhibited a notable reduction in glucose levels (145.5 ± 6.3 mg/dL) compared to the HFD/STZ group. In the IPE-treated groups, fasting glucose levels decreased in a dose-dependent manner. The high-dose IPE group (400 mg/kg) displayed the most pronounced reduction, with glucose levels recorded at 115.25 ± 25.1 mg/dL, even lower than those in the positive control group (Figure 7). These findings suggest that IPE effectively lowers blood glucose levels, particularly at higher doses.

In addition to its effect on blood glucose levels, IPE improves insulin secretion capacity. The HFD/STZ group demonstrated significantly lower serum insulin levels (2.75 ± 0.09 pg/mL) than the NFD group (3.23 ± 0.17 pg/mL), indicating impaired insulin secretion. In the positive control group, insulin levels increased to 2.94 ± 0.24 pg/mL, reflecting partial recovery of insulin function. Similarly, IPE administration resulted in dose-dependent improvements in insulin levels, with values of 2.87 ± 0.27, 2.83 ± 0.27, and 2.82 ± 0.12 pg/mL for the high, medium, and low IPE doses, respectively. These levels were higher than those observed in the HFD/STZ group, further supporting the benefits of IPE in enhancing insulin secretion.

In summary, IPE significantly reduced fasting blood glucose levels and improved insulin secretion in a dose-dependent manner in an HFD/STZ-induced diabetic rat model. These findings highlight the potential of IPE as a therapeutic agent for managing hyperglycemia and insulin dysfunction in diabetes.

### 3.7. Effect of IPE on Phosphorylation of IRS-1 in Pancreas Tissue

**Figure 8:**
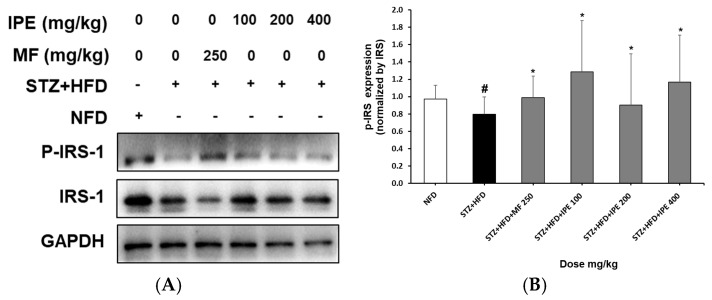
Effect of IPE on phosphorylation of IRS-1 in HFD/STZ-induced diabetic rats. (**A**) Western blots. (**B**) Quantitative graph of western blots. NFD, normal-fat diet; STZ + HFD, streptozotocin + high-fat diet; STZ + HFD + MF, streptozotocin + high-fat diet + diabetic control + metformin. Values are expressed as means ± SEM. ^#^ *p* < 0.01 com-pared with NFD and * *p* < 0.05 compared with STZ + HFD.

The effect of orally administered IPE on phosphorylation of insulin receptor substrate-1 (P-IRS-1) in the pancreas of HFD/STZ rats was investigated. In the HFD/STZ group, the expression of P-IRS-1 was reduced by 0.2 ± 0.2 times compared to the NFD group. In the positive control group, P-IRS-1 expression was increased by 0.2 ± 0.8 times compared to the HFD/STZ group. In the IPE-treated groups, the P-IRS-1 expression increased by 0.5 ± 0.4, 0.1 ± 0.4, and 0.4 ± 0.3 times for the 100, 200, and 400 mg/kg doses, respectively (Figure 8).

## 4. Discussion

Among the several ingredients of IPE, one of the primary active components responsible for the biological actions of IPE is D-pinitol [21]. D-pinitol has been shown to have various anti-inflammatory, anti-cancer, and antioxidant qualities; such qualities have been extensively researched in the context of diabetes [6,8]. However, the mechanisms by which IPE and D-pinitol affect GSIS in vitro are yet unknown. The effect of IPE and D-pinitol on insulin secretion in vitro was examined in this study, along with the possibility that the PDX-1 pathway plays a role in the insulin secretion action of these substances.

Previous studies have demonstrated the positive impact of IPE and D-pinitol on insulin secretion in diabetic animal models [6,8,10,11,12]. Consistently with previous studies, we found that treatment with IPE and D-pinitol increased insulin secretion in INS-1 cells. In contrast, it was reported that D-pinitol from *Ceratonia siliqua* decreased GSIS in INS-1 cells [22]. The differences noted in our study may be due to the variation in exposure times to glucose and D-pinitol, because insulin-secreting β-cells may change their function when exposed to high glucose concentrations for an extended period of time [23]. Research on how D-pinitol affects insulin secretion has produced conflicting findings. Only a few studies have demonstrated that D-pinitol decreases insulin secretion, whereas most investigations have shown that it increases insulin secretion in various experimental models. Similar findings were observed in the present study [6,8,10,11,12].

We performed mechanistic experiments using Western blotting to examine the potential mechanisms underlying the in vitro effects of IPE and D-pinitol on GSIS. Artificially or spontaneously produced reduced expression of PDX-1 in pancreatic cell lines affects GSIS, suggesting that PDX-1 functions as a gene required for GSIS [17]. Given the significance of PDX-1 in islet formation and insulin gene transcription, it is plausible that insulin gene transcription is decreased by PDX-1 deficiency, which in turn reduces insulin production [24]. Therefore, we examined whether this was related to the effects of IPE and D-pinitol on INS-1 cells. In cells following IPE and D-pinitol treatment, PDX-1 expression was much higher than in control cells in this study.

Based on our observations, PDX-1 could be directly or indirectly targeted by D-pinitol or IPE. An upstream PI3K/Akt signaling pathway mediates PDX-1 [25]. A previous study indicated that increased insulin secretion is the outcome of activating PI3K/Akt signaling, which controls the postnatal proliferation and size of individual pancreatic β-cells. PI3K/Akt signaling is facilitated by IRS-2, an upstream activator [26]. Thus, the overexpression of PDX-1 via the IRS-2/PI3K/Akt pathway could upregulate insulin secretion, which might be one of the primary insulin secretion mechanisms. Consistently with prior research, this study found that following IPE and D-pinitol treatment, there was a significant increase in the expression of IRS-2, PI3K, and Akt compared to control cells. However, further research is required to fully understand how IPE and D-pinitol regulate the expression of PDX-1 via its upstream activators, including IRS-2, PI3K, and Akt. Therefore, it is conceivable that IPE and D-pinitol attenuate hyperglucagonemia by increasing insulin secretion, partially through the overexpression of PDX-1 via the IRS-2/PI3K/Akt pathway. These findings suggest that diabetes can be effectively treated with these benefits of IPE and D-pinitol, as there is a lower chance of hypoglycemia.

An HFD induces adiposity and insulin resistance, both of which are prominent features of T2D [25]. In the present study, IPE administration mitigated the excessive weight gain observed in the diabetic models, potentially through improved metabolic regulation. These findings align with previous studies demonstrating the efficacy of certain plant extracts in reducing weight and improving metabolic profiles [26]. However, we found that weight gain was reduced only marginally at certain doses of IPE. Therefore, it is not clear from this study whether the weight improvement was due to the glucose-lowering effect of IPE. From a scientific standpoint, previous research has shown that T2D is recognized to be associated with obesity, but there is little information on how weight changes relate to the risk of diabetes [27]. The results of the OGTTs showed a notable improvement in glucose tolerance in the IPE-treated HFD/STZ models, as evidenced by a reduction in the OGTT-AUC. This suggests that IPE enhances insulin signaling and glucose metabolism, likely by modulating pathways associated with insulin sensitivity [28]. Furthermore, IPE treatment significantly reduced serum ALT, AST, triglyceride, and total cholesterol levels, highlighting its liver-function-ameliorating and lipid-lowering properties. These results suggest that IPE may mitigate the metabolic complications associated with diabetes [29]. One of the critical mechanisms underlying insulin resistance is the impairment of insulin signaling via IRS-1 [30]. IPE treatment increased IRS-1 phosphorylation, indicating the restoration of insulin signaling. This restoration is essential to improve glucose metabolism and insulin sensitivity. These findings support the idea that plant-based extracts can regulate and enhance metabolic functions by modulating key signaling pathways [31,32].

Despite the beneficial effects and mechanisms that we observed, our study had some limitations. This study used the HFD/STZ model, but did not fully replicate all the characteristics of T2D, indicating the need for further animal model studies. In addition, the molecular mechanisms underlying the insulin-secretion-promoting effects of IPE and D-pinitol have not been fully elucidated and require further investigation. In pancreatic cells, glucose transporter 2 plays a significant role through the adenosine triphosphate (ATP)-sensitive, potassium channels-dependent, classical insulin secretion route. The metabolism of glucose increases the ATP/adenosine diphosphate (ADP) ratio within β-cells. Higher ATP levels cause ATP-sensitive potassium channels to close. Therefore, future studies should further explore the potential mechanisms underlying the insulin secretagogue effects of IPE and D-pinitol. The lack of animal and clinical data is a limitation of our study, but it provides an important basis for future research.

## 5. Conclusions

The effects of IPE and one of its active compounds, D-pinitol, on insulin secretion are mediated by the upregulation of IRS-2, PI3K, Akt, and PDX-1 expression. More scientific investigation is required to support the beneficial use of D-pinitol as a novel anti-diabetic molecule. Additionally, we demonstrated its ability to reduce weight gain, improve glucose tolerance, and regulate serum lipid levels. Henceforth, the anti-diabetic action of IPE is partially verified by our scientific evidence, confirming that its efficiency is due to its active compounds, such as D-pinitol. 

## Figures and Tables

**Table 1:** Effect of IPE on the AUC for the OGTT.

AUC				
Group	Interval			
	0–30 min	30–60 min	60–120 min	Total
NFD	102.8 ± 4.3	113.8 ± 6.5	106.4 ± 7.6	323.0 ± 18.4
STZ + HFD	117.3 ± 7.9	137.5 ± 11.7	128.7 ± 8.3	383.5 ± 27.9 ^#^
STZ + HFD + MF 250	110.9 ± 8.4	127.6 ± 7.4	116.5 ± 6.6	355.0 ± 22.4 *
STZ + HFD + IPE 100	118.3 ± 6.2	135.4 ± 4.5	122.3 ± 6.8	376.0 ± 17.6 *
STZ + HFD + IPE 200	117.3 ± 7.1	132.9 ± 10.3	123.4 ± 7.5	373.6 ± 24.9 *
STZ + HFD + IPE 400	115.1 ± 7.4	131.8 ± 12.6	119.4 ± 12.3	366.4 ± 32.3 *

Values are expressed as means ± SEM. ^#^ *p* < 0.001 compared to the normal-fat diet (NFD) group. * *p* < 0.001 compared to the high-fat diet (HFD) + streptozotocin (STZ) group. MF, metformin.

## Data Availability

The original contributions presented in this study are included in the article. Further inquiries can be directed to the corresponding author.

## References

[1] Kim Y.J., Kim H.M., Kim H.M., Lee H.R., Jeong B.R., Lee H.-J., Kim H.-J., Hwang S.J. (2021). Growth and phytochemicals of ice plant (*Mesembryanthemum crystallinum* L.) as affected by various combined ratios of red and blue LEDs in a closed-type plant production system. J. Appl. Res. Med. Aromat. Plants.

[2] Kang S., Kim S., Ha S., Lee C., Nam S. (2016). Biochemical components and physiological activities of ice plant (*Mesembryanthemum crystallinum*). J. Korean Soc. Food Sci. Nutr..

[3] Lee C.-D., Lee H.-D., Kim H.A., Park S.-M., Lee S. (2024). Validation of an HPLC-RI method for the quantification of D-pinitol from *Mesembryanthemum crystallinum*. J. Appl. Biol. Chem..

[4] Rengarajan T., Nandakumar N., Rajendran P., Ganesh M.K., Balasubramanian M.P., Nishigaki I. (2015). D-pinitol mitigates tumor growth by modulating interleukins and hormones and induces apoptosis in rat breast carcinogenesis through inhibition of NF-κB. J. Physiol. Biochem..

[5] Alonso-Castro A.J., Alba-Betancourt C., Rocha-González E., Ruiz-Arredondo A., Zapata-Morales J.R., Gasca-Martínez D., Pérez-Gutiérrez S. (2019). Neuropharmacological effects of d-pinitol and its possible mechanisms of action. J. Food Biochem..

[6] Pandi A., Kalappan V.M., Chandrashekar N. (2022). Effects of D-pinitol on diabetes mellitus: An updated review. Bull. Natl. Res. Cent..

[7] Lee B.H., Lee C.C., Wu S.C. (2014). Ice plant (*Mesembryanthemum crystallinum*) improves hyperglycaemia and memory impairments in a Wistar rat model of streptozotocin-induced diabetes. J. Sci. Food Agric..

[8] Gao Y., Zhang M., Wu T., Xu M., Cai H., Zhang Z. (2015). Effects of D-pinitol on insulin resistance through the PI3K/Akt signaling pathway in type 2 diabetes mellitus rats. J. Agric. Food Chem..

[9] Bates S.H., Jones R.B., Bailey C.J. (2000). Insulin-like effect of pinitol. Br. J. Pharmacol..

[10] Silva J.A., Silva A.C.D., Figueiredo L.S., Araujo T.R., Freitas I.N., Carneiro E.M., Ribeiro E.S., Ribeiro R.A. (2020). D-Pinitol increases insulin secretion and regulates hepatic lipid metabolism in Msg-obese mice. An. Acad. Bras. Ciências.

[11] Navarro J.A., Díaz C., Decara J., Medina-Vera D., Lopez-Gambero A.J., Suarez J., Pavón F.J., Serrano A., Vargas A., Gavito A.L. (2022). Pharmacokinetics and endocrine effects of an oral dose of D-Pinitol in human fasting healthy volunteers. Nutrients.

[12] Zhang C., Wu W., Xin X., Li X., Liu D. (2019). Extract of ice plant (*Mesembryanthemum crystallinum*) ameliorates hyperglycemia and modulates the gut microbiota composition in type 2 diabetic Goto-Kakizaki rats. Food Funct..

[13] Guillausseau P.-J., Meas T., Virally M., Laloi-Michelin M., Médeau V., Kevorkian J.-P. (2008). Abnormalities in insulin secretion in type 2 diabetes mellitus. Diabetes Metab. J..

[14] Modi P. (2007). Diabetes beyond insulin: Review of new drugs for treatment of diabetes mellitus. Curr. Drug Discov. Technol..

[15] Jugran A.K., Rawat S., Devkota H.P., Bhatt I.D., Rawal R.S. (2021). Diabetes and plant-derived natural products: From ethnopharmacological approaches to their potential for modern drug discovery and development. Phytother. Res..

[16] Rinaldi S., Campbell E.E., Fournier J., O’Connor C., Madill J. (2016). A comprehensive review of the literature supporting recommendations from the Canadian Diabetes Association for the use of a plant-based diet for management of type 2 diabetes. Can. J. Diabetes.

[17] Brissova M., Shiota M., Nicholson W.E., Gannon M., Knobel S.M., Piston D.W., Wright C.V., Powers A.C. (2002). Reduction in pancreatic transcription factor PDX-1 impairs glucose-stimulated insulin secretion. J. Biol. Chem..

[18] Wang H., Iezzi M., Theander S., Antinozzi P., Gauthier B., Halban P., Wollheim C. (2005). Suppression of Pdx-1 perturbs proinsulin processing, insulin secretion and GLP-1 signalling in INS-1 cells. Diabetologia.

[19] Lee D., Baek J.Y., Choi Y.J., Han M.J., Kim S.H., Kim T.H., Lee S., Kang K.S. (2024). Glucose-lowering effect of Reducose® enriched with 1-deoxynojirimycin and l-leucine: Studies on insulin secretion in INS-1 cells and reduction of blood glucose in diabetic rats. Heliyon.

[20] Lee D., Kim J., Choi S., Choi J., Lee J.W., Kang K.S., Shim S.H. (2023). 2, 3-Dihydrosorbicillin and chrysopanol stimulate insulin secretion in INS-1 cells. Bioorganic Med. Chem. Lett..

[21] Agarie S., Kawaguchi A., Kodera A., Sunagawa H., Kojima H., Nose A., Nakahara T. (2009). Potential of the common ice plant, *Mesembryanthemum crystallinum* as a new high-functional food as evaluated by polyol accumulation. Plant Prod. Sci..

[22] Navarro J.A., Decara J., Medina-Vera D., Tovar R., Suarez J., Pavón J., Serrano A., Vida M., Gutierrez-Adan A., Sanjuan C. (2020). D-Pinitol from Ceratonia siliqua is an orally active natural inositol that reduces pancreas insulin secretion and increases circulating ghrelin levels in Wistar rats. Nutrients.

[23] Kim W.-H., Lee J.W., Suh Y.H., Hong S.H., Choi J.S., Lim J.H., Song J.H., Gao B., Jung M.H. (2005). Exposure to chronic high glucose induces β-cell apoptosis through decreased interaction of glucokinase with mitochondria: Downregulation of glucokinase in pancreatic β-cells. Diabetes.

[24] Fujimoto K., Polonsky K.S. (2009). Pdx1 and other factors that regulate pancreatic β-cell survival. Diabetes Obes. Metab..

[25] Xiang H., Lei H., Liu Z., Liu Y., Li Y., Qiu Y., Xu L. (2021). Network pharmacology and molecular docking analysis on molecular targets: Mechanisms of baicalin and baicalein against hyperuricemic nephropathy. Toxicol. Appl. Pharmacol..

[26] Liu S., Li X., Wu Y., Duan R., Zhang J., Du F., Zhang Q., Li Y., Li N. (2017). Effects of vaspin on pancreatic β cell secretion via PI3K/Akt and NF-κB signaling pathways. PLoS ONE.

[27] Kim E.S., Jeong J.S., Han K., Kim M.K., Lee S.H., Park Y.M., Baek K.H., Moon S.D., Han J.H., Song K.H. (2018). Impact of weight changes on the incidence of diabetes mellitus: A Korean nationwide cohort study. Sci. Rep..

[28] Cheng Y., Zhang L., Liu P., Sun H. (2020). Herbal Extracts as Modulators of Glucose and Lipid Metabolism: Focus on Hepatoprotection. Plants.

[29] Wang Z., Li H., Dong Y., Hu M., Wang X. (2018). Hepatic Protection Mechanisms of Antidiabetic Agents in High-Fat Diet Models: A Comprehensive Review. Cells.

[30] Lee D., Choi S., Kang K.S. (2023). Protopanaxadiol ameliorates palmitate-induced lipotoxicity and pancreatic beta-cell dysfunction in INS-1 cells. J. Ginseng Res..

[31] Lee W.-Y., Lee C.-Y., Kim C.-E. (2023). Predicting activatory and inhibitory drug-target interactions based on structural compound representations and genetically perturbed transcriptomes. PLoS ONE.

[32] Zhou X., Yang J., He Y., Wang W., Li J. (2020). Plant-Based Therapies and Their Effects on Insulin Secretion and Pancreatic β-Cell Function in Type 2 Diabetes. Antioxidants.

